# EEG-Based Brain–Computer Interfaces for Communication and Rehabilitation of People with Motor Impairment: *A Novel Approach of the 21^*st*^ Century*

**DOI:** 10.3389/fnhum.2018.00014

**Published:** 2018-01-31

**Authors:** Ioulietta Lazarou, Spiros Nikolopoulos, Panagiotis C. Petrantonakis, Ioannis Kompatsiaris, Magda Tsolaki

**Affiliations:** ^1^Information Technologies Institute, Centre for Research and Technology Hellas, Thessaloniki, Greece; ^2^1st Department of Neurology, University Hospital “AHEPA”, School of Medicine, Aristotle University of Thessaloniki, Thessaloniki, Greece; ^3^Greek Association of Alzheimer’s Disease and Related Disorders, Thessaloniki, Greece

**Keywords:** brain–computer interfaces (BCI), electroencephalogram (EEG), slow cortical potentials (SCP), sensorimotor rhythms (SMR), P300, communication, rehabilitation, neuromuscular disorders (NMD)

## Abstract

People with severe neurological impairments face many challenges in sensorimotor functions and communication with the environment; therefore they have increased demand for advanced, adaptive and personalized rehabilitation. During the last several decades, numerous studies have developed brain–computer interfaces (BCIs) with the goals ranging from providing means of communication to functional rehabilitation. Here we review the research on non-invasive, electroencephalography (EEG)-based BCI systems for communication and rehabilitation. We focus on the approaches intended to help severely paralyzed and locked-in patients regain communication using three different BCI modalities: slow cortical potentials, sensorimotor rhythms and P300 potentials, as operational mechanisms. We also review BCI systems for restoration of motor function in patients with spinal cord injury and chronic stroke. We discuss the advantages and limitations of these approaches and the challenges that need to be addressed in the future.

## Introduction

Vidal (1973, p. 157), in his seminal work, raised the question: “Can observable electrical brain signals be put to work as carriers of information in person–computer communication or for the purpose of controlling devices such as prostheses?”. Since then, we have come a long way investigating whether people with motor disabilities can repurpose brain activity from inner neural signals to tangible controls that attribute the user’s intent to interact with devices or adjust their environment (Shih et al., 2012; Lebedev and Nicolelis, 2017). Nowadays, several advancements in the fields of clinical neurophysiology and computational neuroscience have led to the development of promising approaches based on non-invasive BCIs that pave the way for reliable communication and effective rehabilitation of people with disabilities.

**FIGURE 1:**
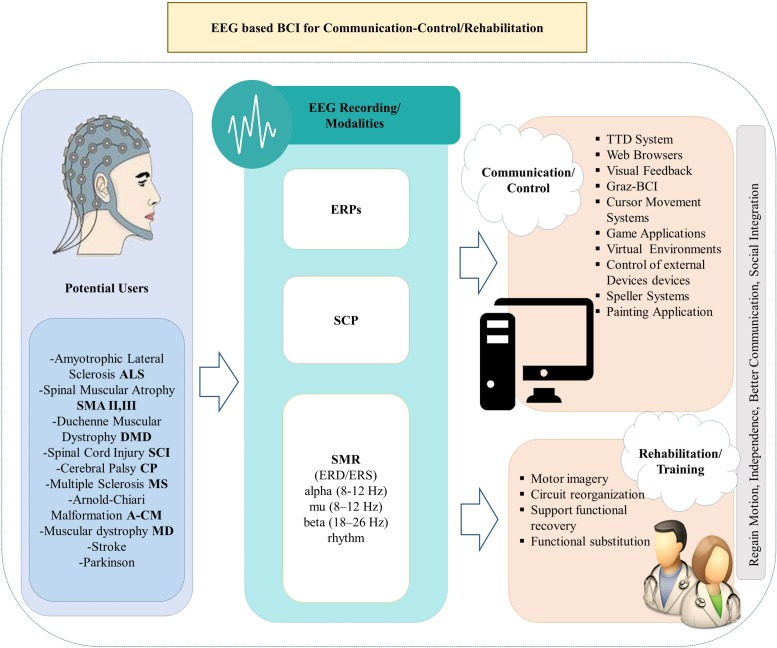
Overview of the review framework.

In this review, we focus on non-invasive BCI applications geared toward alternative communication and restoration of movement to paralyzed patients. We consider several milestone studies on EEG-based BCIs that contributed to the systems that improve everyday life and activity of people with motor disabilities in the 21^st^ century. We review EEG-based BCI technologies for communication and control based on three different EEG signals (SCP, SMR and P300), and discuss their limitations and advantages. In addition, we examine and analyze the BCI methods for inducing brain plasticity and restoring functions in impaired patients. An overview of the study framework is presented in **Figure 1**.

The paper is structured as follows. We firstly review the advantages of the BCI approach compared to other strategies for communication in people with motor impairment. In section 3, we present BCI realizations based on different approaches for brain activity recording, and elaborate on three EEG-based modalities: SCP, SMR, and P300. Subsequently, we provide an elaborate overview of the milestone studies, published mainly during the last two decades, on the BCI systems for communication and rehabilitation in patients with motor-impairments. Finally, we discuss advantages and shortfalls of these BCIs, point out their limitations and comment on the future perspectives in this field.

## Why Brain–Computer Interfaces?

In most cases of NMD, there is gradual loss of muscle activity that affects speaking, walking and execution of fine motor tasks and results in the deterioration of quality of life. Although the symptoms of the diseases are easier to cope with during their early stages, they become much more severe in the late stages. Thus, specific priorities must be set forth in order to develop systems that will be deployable for each stage of the disease, even for late stages. The majority of people with NMD indicate a preference for portable solutions, such as a tablet or a laptop (Mackenzie et al., 2016). Nevertheless, one common issue that may arise of these devices, is their inability to adapt to patient’s need, especially at late stages of the disease where many abilities have deteriorated. Even though many technological adaptive devices are currently utilized, such as “SmartNav^1^” and eye gaze technology, they require extensive user training and have several limitations (Mackenzie et al., 2016). For example, the gaze-based technology has been found to be erroneous for selecting small objects/icons, burdensome and difficult to be operated, difficult to be adjusted and calibrated, as well as costly (Ball et al., 2010).

In addition, current technological assistive solutions using eye trackers enable people with motor disabilities to communicate and control devices at home. The abovementioned systems result in a very low workload (Calvo et al., 2008), whereas participants with ALS have responded positively to using them on a daily basis (Gibbons and Beneteau, 2010). Nevertheless, the main problem of using eye trackers as an assistive technology (AT), is the so-called “Midas touch problem” (Majaranta and Räihä, 2002). In essence, the direction of gaze in many cases does not coincide with the focus of attention and consequently the final selections are different from the users’ intention (Majaranta and Räihä, 2002; Karat and Vanderdonckt, 2009; Nicolas-Alonso and Gomez-Gil, 2012; Pasqualotto et al., 2015; Ordikhani-Seyedlar et al., 2016). Other systems (e.g., “Eye-Go”) utilize eye-tracking methods for wheelchair maneuvering (Gajwani and Chhabria, 2010). Nevertheless, there are still several limitations regarding both the ability to alter or change seating position and the capacity of maneuvering the wheelchair to a specific direction by using eye movement, which poses requirements for both system software and for eye-tracking technology (Wastlund et al., 2015).

Besides gaze-based interaction, there are also speech-based interfaces like the Dragon Dictate Speech (DDS) recognition system (Zimmer et al., 1991). The system learns a user’s vocabulary and mode of speaking by analyzing language and responds to natural language instead of limited phrases and words. However, it needs many adaptations, such as microphone positioning, and training, so it is not useful for people at late stages of NMD who have totally lost their ability to speak. Furthermore, speech-driven interfaces are limited by the number of supported languages. Although English is extensively supported, this is not the case for less popular languages (e.g., Greek).

Other solutions utilize robotic assistive devices for motor disabled people (Corke, 2007). The usability of such devices is still doubtful due to the necessity of residual motor ability for their operation (e.g., limb, head and/or eye movements, speech etc.) (Cincotti et al., 2008b). For people in extreme pathological conditions (e.g., those who are in CLIS without any remaining muscle control), the use of such systems may not be possible.

Additionally, recent studies have underlined the importance of BCI systems in rehabilitation and restoration of motor function. For instance, the Personalized Self-Managed rehabilitation System (PSMrS), is a prototype system integrated with an insole sensor technology (“intelligent shoe”) (Mawson et al., 2016). It has been developed to enable people who survived stroke to self-manage their rehabilitation by encouraging achievement of personal and adaptive functional goals and performance of specific motor activities within those goals. However, people who participated in this study and used this technology reported a number of limitations such as the lack of feedback and lack of motivation of the user (Mawson et al., 2016). Other studies focus on the use of technical solutions for the rehabilitation of participants with motor disabilities (e.g., MS), by using KINECT and augmented reality (AR) for rehabilitation of gait (Lozano-Quilis, 2013; McMullen et al., 2014), or the use of natural user interfaces (NUIs) for rehabilitation of participants with reduced motor skills (Fulk, 2005; Baram and Miller, 2006; Lozano-Quilis et al., 2014). Systems for motor rehabilitation of MS participants were tested with patient in the mild stages of the disease (they were capable to move their limbs), but their effectiveness in the late stages (e.g., LIS) has not been documented.

Brain–computer interface systems promise to offer a unique and multimodal solution for both communication and rehabilitative therapy that will overcome the shortfalls of the abovementioned approaches (Chaudhary et al., 2016). In general, BCI is a computer-based system which translates brain signals into commands that are passed onto an external application or appliance so as to facilitate user’s intention. As a result people can communicate with the environment, although they do not use their peripheral nervous system and muscles (Wolpaw et al., 2000). The idea of operating an external device with one’s thoughts is a highly promising option for people whose functions such as speech or motion are impaired. Many clinical studies on BCI research field have highlighted not only the potential utility and integration of these innovative technological approaches into life of people with motor impairment, but also the positive impact occurring by translating scientific knowledge and experimental design into clinical benefits by enabling a novel real-time communication between the user and the external world (Bowsher et al., 2016). In terms of utility, BCI technology gives the opportunity to individuals who are not able to speak and/or use their limbs not only to communicate and interact with others, but also to operate such appliances as domestic devices, computers, speech synthesizers and assistive deployments, or even control neural prosthetics for walking and manipulating objects (Wolpaw et al., 2002); even in CLIS (Chaudhary et al., 2017). Nevertheless, many BCI systems have also some drawbacks, since users might need long periods of training to learn to control their brain rhythms (Pires et al., 2012) or have problems to conveniently operate the BCI system such as problems with switching it off and on (“Midas touch” problem) (Nicolas-Alonso and Gomez-Gil, 2012). Nevertheless, BCI systems could improve the quality of life (QoL) of people with motor impairment as a result of social inclusion (Sellers et al., 2010; Holz et al., 2015), even in CLIS (Liu et al., 2011). Indeed, communication ability is of high priority in improving QoL of people with motor impairments (Bach, 1993; Kubler et al., 2005).

## BCI Systems

There are various techniques and operational mechanisms that are used in order to record brain activity and extract useful signals. BCI systems are based on invasive recordings, i.e., implantable electrodes or grid recordings from the cortex (ECoG) (Bleichner et al., 2016), local field potentials (LFPs) (Jerbi et al., 2009), multi-unit activity (MUA) or single-unit activity (SUA) (Watanabe, 2011), as well as non-invasive methods such as EEG, magnetoencephalography (MEG) (Ahn et al., 2013), functional magnetic resonance imaging (fMRI) (Zich et al., 2015), and near-infrared spectroscopy (NIRS) (Hosseini et al., 2016). Other approaches concerning combinations of aforementioned recording techniques with additional electrical stimulation have also been proposed, such as electrical/magnetic stimulation (Daly et al., 2009; Ang et al., 2015; Hanselmann et al., 2015). From all the abovementioned approaches for BCI system realization, EEG recordings have been more thoroughly studied and used (Hinterberger et al., 2003; Klobassa et al., 2009; Corralejo et al., 2014) due to their effective implementations, non-invasiveness, and portable and adaptable manifestation, (Remsik et al., 2016). Thus, this review concerns only EEG-based BCI systems. Moreover, different BCI applications will be reviewed and evaluated qualitatively and quantitatively using two measures: *CA*, i.e., the percentage of correctly classified BCI controls when the user operates a BCI system and *ITR*, i.e., the ITR, given in bits per selection or bits/min. In the case of CA we will use the terms Low or High performance, *with Low performance referring to CA lower than 90%* (Huggins et al., 2011).

### EEG-BCI Neurophysiological Input

There are different types of EEG-based BCI systems for promoting communication (Wolpaw et al., 1991), environmental control and rehabilitation depending on different modalities of the EEG, namely SCPs, SMR, P300 event related potentials and Steady State Visual Evoked Potentials (SSVEPs).

#### Slow Cortical Potential

An EEG-based BCI can rely on SCPs, which allow anatomically specific voluntary activation of different brain areas. Neurophysiologically, SCPs are the result of intracortical or thalamocortical inputs to different cortical layers; they arise from simultaneous polarization of large cohorts of apical dendrites of pyramidal neurons (Kübler and Birbaumer, 2008). Depolarization of cortical cell assemblies reduces their excitation threshold, and firing of neurons in regions responsible for motor or cognitive tasks is facilitated (Birbaumer et al., 1990). In normal brain function, negative SCPs accompany preparatory depolarization of the underlying cortical network, whereas positive SCPs are thought to reflect cortical disaffiliation or inhibition. With substantial training, control of SCPs to produce positive or negative voltage shifts can be learnt and used for basic word processing and other tasks (Leeb et al., 2015). However, SCP-BCI systems need long training periods, professional attention and continuous technical support, while not all patients can gain full control of their SCPs.

#### Sensorimotor Rhythms

Apart from SCP, there are many EEG-based BCI systems that rely on SMRs (Neuper et al., 2003; Wolpaw, 2007; Sellers et al., 2010). SMRs correspond to rhythms extracted from EEG signals that are recorded exclusively over sensorimotor areas (i.e., μ-rhythm and β-rhythm) and allow anatomically specific voluntary regulation. Typically they are movement-modulated rhythms, i.e., there is a change either while someone performing a movement or during MI or movement execution. Results from BCI studies have shown that people can learn to self-regulate μ-rhythm or β-rhythm amplitudes in the absence of any movement or sensation, and subsequently to move a cursor to a specific position of the computer screen, to select letters, or to operate an orthotic device (Wolpaw and McFarland, 2004). In general, SMR-based control is achieved through activation and deactivation of the central motor areas and no external (e.g., visual) stimuli is necessary, since the oscillatory EEG components are modulated by specific mental strategies (e.g., MI of left hand) (Pfurtscheller and Neuper, 2001).

There are two distinct power changes in SMRs: (i) ERD or power decrease seen in both alpha and beta bands that occur up to 2 s before movement (Pfurtscheller et al., 1998; Bai et al., 2005), and (ii) ERS or power increase, usually seen in the beta band and referred to as post-movement beta synchronization, occurring after the end of a movement (Pfurtscheller, 1988). It has been demonstrated that SMR–based BCI systems are possible to enable people with motor disabilities to operate an environmental control system or a simple word-processor faster than SCP-EEG based BCI systems (Wolpaw and McFarland, 2004; Kübler et al., 2005), while, it has been also proposed as a potential solution for motor rehabilitation (Pfurtscheller et al., 2000; Ono et al., 2014). On the contrary, the need for extensive training sessions that are necessary to gain sufficient SMR regulation control, comprise the main shortfall of this approach. Finally, another drawback of the SMR approach is the discrimination capacity of SMR features that correspond to different imaginary movements that in some cases results in ineffective communication (Karat and Vanderdonckt, 2009).

#### Evoked Potentials

P300 is an ERP and is one of the most commonly used EEG-based modalities (Donchin, 1987). The P300 is evoked approximately 300 ms after a triggering stimulus; it corresponds to a positive voltage peak (Li et al., 2010). P300-based BCIs capitalize on discriminating this voltage peak from background signal noise, and associating the desired signal with an event. ERPs are associated with a stimuli that provides task-relevant information (Baykara et al., 2015). P300 has been popular in BCI research due to its ease of evocation and consistency (Duncan et al., 2009). It requires little initial training of the subject (Piccione et al., 2006; Sellers and Donchin, 2006; Fazel-Rezai et al., 2012). Nevertheless, in a recent meta-analysis (Marchetti et al., 2014), accumulated evidence from recent studies of P300-speller BCIs with ALS participants, showed that the estimated CA was of the order of 74%, much lower than 90% that has been set as the acceptable threshold for ALS participants (Huggins et al., 2011). Moreover, it has been shown that P300-based BCI systems could be affected by overall clinical severity (Piccione et al., 2006; Sellers and Donchin, 2006; Hoffmann et al., 2008) and worse BCI performance can be detected in motor impaired ALS patients due to habituation or fatigue effects (Sellers and Donchin, 2006). Recent studies suggested that P300-based BCIs could be useful for ALS patients (Nijboer et al., 2008; Corralejo et al., 2014). Finally, some studies reported as main disadvantage the low ITR of P300-based BCIs, especially for people with motor impairments (Mugler et al., 2010; Mauro et al., 2011; Nam et al., 2012; Holz et al., 2013). Nevertheless, higher ITRs have also been reported (Kaufmann et al., 2013) using appropriately selected stimuli (e.g., faces).

Steady State Visual Evoked Potentials are evoked by an oscillating stimulus modulated at a fixed frequency and occur as an increase in EEG activity at the stimulation frequency (Valbuena et al., 2010). The typical SSVEP-based BCI application utilizes multiple visual flashing stimuli, such as digits or letters on a screen, while the user looks at one of the symbols and focuses his attention on it (Lesenfants et al., 2014). When the users concentrate their attention on one of the stimuli, the stimulus evokes an increased SSVEP response at the corresponding frequency over the occipital area of the brain. Thus, the user has to keep eye fixation. This type of BCI depends on attentional capacity and vision to be intact, and both are often compromised in patients with advanced and severe neurological disease (Chaudhary et al., 2016). Therefore, SSVEPs are not suitable for patients in advanced stages of Amyotrophic Lateral Sclerosis (ALS-LIS) or with uncontrollable eye movements (Nicolas-Alonso and Gomez-Gil, 2012). A number of SSVEP-based BCI systems have been developed and applied to operating a prosthesis (Muller-Putz and Pfurtscheller, 2008) or controlling an avatar in a virtual reality environment (Faller et al., 2010) by healthy individuals. The conventional SSVEP-based BCI systems described above commonly require the basic assumption that the users have normal oculomotor function and are thus able to maintain gaze at a given visual stimulus consistently (Lim et al., 2013). However, as already mentioned, it has been reported that some patients suffering from serious NMDs have difficulty controlling their eyes (Donaghy et al., 2010), while many ALS patients have oculomotor impairments (Sharma et al., 2011) causing abnormal visual perception. These patients would not be able to open their eyes and continuously fixate a visual stimuli. A recent study used SSVEP-BCI in ALS patients with LIS and only one out of four was able to communicate online. This low success rate was attributed to symptoms accompanied with clinical conditions of these patients: fatigue, persistent nystagmus preventing effective perception of the stimuli (Lesenfants et al., 2014). This represents an important limitation for patients with loss of gaze control and other clinical issues. For all the aforementioned drawbacks, in this work we exclude the SSVEP studies as we intend to discuss approaches concerning users with motor impairments.

## EEG-Based BCI Systems For People With Motor-Impairment

Electroencephalography-based BCI systems show great potential for widespread clinical use. Over the past two decades, many studies have been published presenting non-invasive approaches for severely or partially paralyzed people reacquiring basic forms of controlling neuroprostheses, wheelchairs and electrical appliances. The purpose of using BCIs can be distinguished in two main categories: (a) Communication and Control and (b) Rehabilitation. By *communication and control* we refer to the ability of BCIs to enable communication with such devices as speech synthesizers, word processors, computer email applications, wheelchairs, prosthetic robotic arms, or orthotic devices. (Control of a device can be considered as a means of communication with the environment, and thus we consider the term “communication” in its generic meaning in this review). Finally, by *rehabilitation* we refer to therapies that use BCI systems for regaining the lost motor control for people suffering from neurological disorders (e.g., stroke). Below, we elaborate on these concepts.

### Communication and Control

#### BCI Systems Using Slow Cortical Potentials (SCP)

##### The TTD system

Slow cortical potentials-EEG-based BCI systems require users’ training to shift the polarity (positive or negative) of their SCPs. The first seminal study on EEG-BCI with two patients in LIS ALS using SCP was conducted almost two decades ago (Birbaumer et al., 1999). The patients were trained to voluntarily generate SCPs, lasting 2–4 s, and during the response period, the subjects were required to produce either negativity or positivity greater than a specific amplitude. In a subsequent work (Birbaumer et al., 2000) a TTD was developed. TTD trains participants at LIS to self-regulate their SCPs in order to select letters, words or pictograms in a computerized language support program. This was one of the first studies to show potential usefulness and crucial role of TTD system in everyday life while great improvement was shown regarding self-regulation and correct responses after long period of training of five participants (LIS). Kubler et al. (2001) expanded the previous TTD system by introducing for the first time “feedback training” by testing two male participants at late ALS stage. The participants underwent a training procedure which enabled them to communicate verbally, by self-controlling their SCPs within the “Language Support system” (Birbaumer et al., 2000), i.e., a system where the alphabet is split into halves (letter-banks) which are presented successively at the bottom of the screen for several seconds. If the subject selects the letter-bank being shown by generating an SCP shift, the letter bank is then split into two new halves and so on, until each of the two letter-banks has only one letter in it. After a training procedure of several months severely paralyzed participants were able to communicate verbally. Moreover in another work (Hinterberger et al., 2003), they trained an ALS patient to use TTD for over 1 year so as to use this device in order to communicate by spelling words. Also, they compared different EEG classification algorithms of the three phases of training in two sets (Set 1:“feedback training” phase by rewarding the patient for producing cortical shifts in a requested direction, Set 2: “copy-spelling mode” by requiring the participant to copy a text, and “free-spelling mode” by self-selecting letters and words). The patient’s average CRRs in the on-line training were 83 and 72% for the Set 1 and Set 2, respectively.

In another work, five ALS participants were trained on how to use their SCPs for effective communication. Particularly, the procedure included a training phase while during the copy spelling and free spelling mode, participants had to select a letter by producing positive SCP amplitude shifts or to reject a letter by producing negative SCP amplitude shifts (Neumann and Birbaumer, 2003). After several training sessions, they measured the CRR and found that the more the participants attended training sessions the higher was the accuracy and correct response of each individual (except for one subject who stopped the training). In essence, it was demonstrated that the performance in SCP self-regulation after several sessions of training can be predicted from an initial performance.

Another approach investigated the potential use of SCPs as a control mechanism in order to promote communication of an ALS patient by providing visual feedback of his actual SCP amplitude (Neumann et al., 2003). More specifically the experiment contained two training phases: (i) *basic training:* the participant was trained in order to regulate his SCP amplitude to exceed or to remain below a certain threshold in the cursor movement time of the active phase, while in (ii) *letter production training*: the participant had to “select” or “reject” letters by self-regulating his SCPs. In general, this was a strategy for the participants with motor impairment to learn how to write a text based on his mental strategies to move the cursor (basic training). After 6-months of training, the participant managed to self-regulate his SCP by producing two different brain responses. At the end he managed to produce 454 words in German language even though the speller yielded one letter per minute (a complete text written by the participant using self-regulation of SCPs is presented in the published article). Nevertheless, the participant responded positively regarding the system’s use (Neumann et al., 2003).

##### Web browsers

Another approach to using TTD system for handling a Web Browser, namely “Descartes,” was firstly introduced in Karim et al. (2006). The embedded system was tested with only one ALS patient. Descartes provided feedback of SCP amplitude time-locked manner after several sessions of training. To select a command, the participant had to produce positive SCP alteration (above a threshold of 7 μV) and move the cursor downward, while for discarding a command, the participant had to generate negative SCP and thus move the cursor upward. This was the first study to show that an EEG-controlled Web browser, which is based on SCPs self-regulation, could be efficiently and successfully operated by a severely paralyzed patient. It was possible to access internet via the “Descartes” system but there was great difficulty as regards to selecting an icon or a picture on a Web page or selecting from an alphabetically sorted decision tree. These disadvantages raised some issues regarding the everyday use of such systems. Although the training phase of the aforementioned study may be a proper procedure in order to support patients so as to take advantage of BCIs and foster communication, the participant had to exceed or to remain below a certain threshold (7.7 μV) for operating the system and this containment may not be suitable for everyday use.

#### BCIs Using Sensorimotor Rhythm (SMR)

People with NMDs can also learn to modulate their SMR generated when a specific movement is executed or simply imagined (MI).

##### Graz BCI system

One of the most highlighted SMR-BCI studies, namely the “Graz-BCI” was tested on a severely paralyzed participant with CP, who had totally lost the ability to communicate (Neuper et al., 2003). After 80 trials the participant was able to select letters and to copy words by using self-regulation of his SMR. This study showed that “Graz-BCI” could effectively decode changes in SMR due to MI with a CA of 70% in the task of letter selection. Nevertheless, the spelling rate was very low (one letter per minute) and many adaptations of the classifier were necessary. On the other hand, SMR was also successful in many cases with good CA (above 70%) but extensive training and system adaptations were needed in almost all cases (Neuper et al., 2003). Moreover, “Graz-BCI” was also used to enable four paraplegic participants to communicate by using a MI task that also incorporated a feedback module. More specifically, the task in this study was a right-hand movement imagination, the so-called “Basket-paradigm” with feedback, where the participants had to move the ball into the correct “basket” (Krausz et al., 2003). One aspect that should be highlighted in this study was that the participants achieved high CA and an ITR between 8 and 17 bits/min without participants having any experience with the BCI system before. This proved that the “Gratz-BCI” paradigm can be easily learnt even by people who are not skilled at these applications.

##### Cursor movement systems

Similarly, Wolpaw and McFarland (2004) developed an EEG-BCI system based on SMR regulations. In this study the target location was block-randomized (eight blocks) while the cursor was presented in the middle of the screen. The cursor movement was a weighted combination of the amplitudes in a mu (8–12 Hz) or beta (18–26 Hz) rhythm over the right and left sensorimotor cortices. Their results indicated that people can learn to use scalp-recorded EEG rhythms to control movement of a cursor in two dimensions, while this control is developing gradually over training sessions. This study demonstrated that performance gradually improved over the training sessions while participants gradually gained better control over the rhythm amplitudes that controlled the cursor.

In another study (Kübler et al., 2005), four ALS participants were trained to move the cursor steadily across the screen with its vertical movement controlled by SMR amplitude. After a training period of 3–7 months, it was possible by MI to reduce rhythm amplitude and, thus, to move the cursor down, while no-imagery state did the opposite. Their results showed that over the initial 20 sessions of training, all four participants acquired SMR control, proving that a SMR–based BCI might help participants with ALS to maintain an acceptable quality of life with appropriate communication systems (Kübler et al., 2005).

To exploring the feasibility and accuracy of the SMR approach, one recent study (Cincotti et al., 2008a) applied high-resolution electroencephalographic (HREEG) techniques that estimated cortical activity by using appropriate models of volume conduction and neuro-electrical sources. In this study, the authors examined 5 able-bodied participants and one with a traumatic stabilized lesion located at the dorsal level. All of them underwent a series of EEG acquisition sessions, in which they were trained to gain control of their SMR, while they were instructed to concentrate on kinesthetic imagination of upper or lower limb movement in order to move the cursor up-right and down-right, respectively. In this study, the lateralization of electrical activity, which is expected to be contralateral to the imaginary movement, is more evident on the estimated CCD than in the scalp potentials and showed that subjects who underwent training could utilize voluntary modulation of estimated CCDs for accurate on-line control of a cursor.

Furthermore, McFarland et al. (2008) demonstrated remarkable results as people with severe motor disabilities could use brain signals for sequential multidimensional movement, selection with two-dimensional cursor movement and target selection through self-regulation of their SMR. This is one of the first and most highlighted studies which showed that people with motor impairment can learn to use scalp-recorded EEG rhythms so as to move a cursor in two dimensions to reach a target and then to select the target.

##### Game applications

A noteworthy study tried to address the problem of long training periods needed for people with motor impairment to learn how to operate an EEG-BCI system. A game experiment that could achieve a successful BCI operation in less than 30 min was proposed (Kauhanen et al., 2007). More specifically, 6 tetraplegic participants were asked to move a circle (which appeared on the center of the screen) left or right by choosing one of the movements of the upper limbs (e.g., fist closing) and use them (imagery task) during the experiment. Their results indicated that three out of six subjects learned to control a BCI after training and hit the target with rates 2.2–3.8 hits/min, with accuracy of 94, 67, and 57%, respectively, and an ITR of 8 bits/min. They concluded that subjects could improve their performance after more training since they could learn to produce more distinctive brain activations during the attempted movements.

Similarly, Bai et al. (2008) investigated the role of extensive training in the ability to control SMR. This is one of the few studies where people with motor impairment showed equal performance, in terms of CA, with able-bodied ones. It was underlined that the reduced training time may be particularly beneficial for patient populations who have difficulty in sustaining the mental effort and concentration needed for controlling SMR. In particular, a new BCI method was proposed where subjects were asked to sustain motor execution (ERD) or stop movement/imagery (ERS) in a binary cursor-control game. Two motor impaired participants (one with stroke and one with ALS) and nine healthy subjects participated and it was found that by using MI, subjects were able to operate the proposed system with good accuracy and with fast transfer rates (10–12 bits/min) while, in terms of CA, average accuracy was, for motor execution 93.1% (healthy), 83% (Stroke), MI 80% (Stroke) and for ALS: motor execution 90%, MI 90%.

Another study (Holz et al., 2013) developed a different approach for self-control of SMR rhythms throughout a game, namely the “Connect-Four.” The proposed system exhibited low effectiveness, as compared to effectiveness achieved by end-users in other SMR-based BCI systems (70–100%) (Kauhanen et al., 2007).

Although some of the abovementioned studies tried to address SMR based BCI systems by reducing the time needed for training, their results revealed low ITR 2.2–3.8 hits/min and showed that participants’ performance improved with longer training periods (Kauhanen et al., 2007; Jeon and Shin, 2015). This comes to add in the great body of the literature suggesting that training is necessary to gain successful self-control of SMR.

##### Virtual environments

Another study explored how a tetraplegic subject, sitting in a wheelchair, could control his movements in a VE with a self-paced (asynchronous) BCI system (Leeb et al., 2007). The usage of the VE framework with talking avatars ensured that the experiment is novel and engaging and contains enough distractions as it would be in a real street environment. After four runs, the subject was able to reach accuracy of 100%. This work was the first study which included avatars and VE and introduced new ways of interaction and control of applications for people with motor impairment in a VE by using MI. The fact that after only four runs, the subject reached a successful performance of 100% shows that motivation induced by responses of the avatars and more realistic conditions enables the participant to achieve better performance (Leeb et al., 2007).

##### Control of external devices

There are also many studies which have highlighted the importance of EEG-BCI for controlling external devices. In Cincotti et al. (2008b) it was shown that people with severe NMDs can acquire and maintain control over detectable patterns of brain signals, and use this in order to control devices. Subjects were asked to execute or to imagine movements of their hands or feet upon the appearance of respective target. This study showed for the first time that an EEG-based BCI system can be integrated into an environmental control system. If the user was not able to master any of the devices, a training procedure followed. Users needed to learn to modulate their SMR to achieve more robust control than a simple imagination of limb movements can produce. Over the 10 sessions of training, subjects acquired brain control with an average classification higher than 75% in a binary selection task. Nevertheless, it should be stressed out that only four out of 14 participants managed to achieve the abovementioned accuracy.

In another study the potential use of SMR in control of external domestic devices was investigated. As far as the experimental procedure is concerned, initially, a rectangular target appeared on the right side of the screen (up-right or down-right). Afterward a cursor appeared in the middle of the left side of the screen and moved at a constant horizontal speed to the right. During the training sessions, subjects were asked to imagine the same kinesthetic movement during the visual session. Four out of six participants with DMD were able to control several electronic devices in the domestic context with the BCI system, with a percentage of correct responses averaging over 63% whereas healthy subjects achieved rates of 70–80%.

Another study (Conradi et al., 2009), introduced the “one-dimensional feedback task,” i.e., moving a cursor from the center of a monitor to a randomly indicated horizontal direction. In the second stage of the experiment, the feedback signal was provided only at the end of each trial. Four out of seven subjects were able to operate the BCI system via attempted (not imagined) movements with their impaired limbs (both foot and hand) with up to 84% CA. Moreover, it was highlighted that the foot movements were more easily discriminated that hand movements in the majority of the participants.

Furthermore, a recent study demonstrated the potential use of a telepresence robot, which is remotely controlled by a BCI system during a navigation task. In this task the participants with motor impairment achieved similar CA to ten able-bodied participants who were already familiar with the environment (Leeb et al., 2015). The robot starts at a specific position, and there were four target positions to be reached. The user’s task was to drive the robot along one of three possible paths and then back again to the starting position. Some end-users were able to press specific buttons on a modified keyboard while others were using “head switches” by imagining left hand, right hand and feet movements during calibration recordings. However, people with motor impairment needed more time in comparison with healthy participants to complete the path. It is noteworthy that the environment of the experiment contained natural obstacles as in a real-life environment.

#### BCIs Using P300

An EEG modality that is very frequently used for operating a BCI system is the widely known P300 component, which is a late positive component evoked in response to an external task-relevant stimulus. In particular the P300 component has been used in order to control devices, such as wheelchairs, operate real and VEs, and allow the user to use paint interfaces or browse the Internet.

##### Speller systems

Speller systems are the main BCI applications that use the P300 modality. One seminal work (Sellers and Donchin, 2006) investigated whether P300-BCI could be used as an alternative EEG-based BCI modality for communication in ALS population. They evaluated the effectiveness of a BCI system by detecting P300 elicited by one of four randomly presented stimuli (i.e., YES, NO, PASS, END) and by testing this to both able-bodied and ALS participants. The participant’s task was to attend one stimulus (audio, visual, or in both) and reject the others. For instance, one task was to focus on the target stimulus (i.e., YES or NO as defined by the experimenter at the beginning of each run), while the other task was to focus on the stimulus (YES or NO) that correctly answered a question provided by the experimenter. In terms of CA, groups’ performances were similar, which suggests that a P300-based BCI can be considered as a powerful and cost-effective, non-muscular communication tool both for ALS and non-ALS, able-bodied users. However, the ITR (bits/selection) in this study ranged from 0.43 to 1.80 which is considered rather low compared to other similar, more recent P300-based BCI studies (Mugler et al., 2010). In addition, the most severely impaired ALS participants had the worse CA compared with other participants, which reflects also the incapability of P300 to be an effective communication solution for more severe impairments.

In this vein, another study (Hoffmann et al., 2008) developed a P300-based BCI system tested on five people with motor impairment with separate pathologies and four able-bodied subjects. The experimental protocol simulated an environment-control paradigm where images including objects (four electrical devices a door and a window) were flashed in random sequences. Four out of the five disabled participants and two out of the four healthy subjects achieved 100% offline accuracy. Similarly, Nijboer et al. (2008) tested severely disabled ALS individuals on a P300-based BCI system for writing text by using a two-phase experimental procedure. Initially, the subjects had to copy-spell a letter and then free-spell letters by using ‘Space’ button on conclusion/end of a word, ‘Backspace’ button to delete the most recent character selected, and ‘End’ button to finish the run. In copy-spelling sessions, the computer prompts the user with text to copy character by character within 10 sessions by using a matrix-spelling task, where 36 squares contain the alphabet and the numerals 0–9. Then, in Phase II, each subject completed at least 10 free-spelling sessions (choose characters at will). It was shown that ERP response remained stable for several months (no statistical decrease of amplitude or latency of P300 over time). Moreover, CA improved during free-spelling phase.

In another study (Kübler et al., 2009), 4 ALS participants who had already been involved in similar studies, operated a 5 × 5 spelling matrix (all letters of the alphabet, except for letter Z) with auditory feedback. To select one target letter, the participants had to first attend to the number that coded the rows (1–5) and columns (6–10). This study showed extremely low selection and CA (4% for selection accuracy and 20% for CA), which demonstrates that it is exceptionally troublesome for participants to focus and maintain their attention on the numbers, and that the “visual support matrix” is ultimately essential. Another study (Nam et al., 2012) used P300 Speller Paradigm with 7 × 7 matrix of alphanumeric characters, where two types of stimuli are presented with different probability (infrequent target stimuli and frequent non-target stimuli). People with motor impairment exhibited lower CA (i.e., 36%) compared to the able-bodied group (88%). Moreover, the ITR was also lower for the motor disability group (0.65 bits/min) than the able-bodied group (1.96 bits/min).

A more recent approach (Kaufmann et al., 2013) compared the “Classic Flashing (CF)” P300-Speller paradigm, in which rows and columns are highlighted randomly (as in common P300- Spellers) and the “Face Flashing (FF),” in which characters (letter and numbers) are overlaid with faces so as to investigate effects of face familiarity on spelling accuracy. Two motor-impaired participants were not able to communicate with more than 40% CA with CF whereas in FF case same participants spelled with an average accuracy of approximately 82%. These findings show the benefits of the FF case to such a degree that motor-impaired participants’ performance did not significantly differ from motor-able participants. On the other hand, low ITR and CA reported in a study that examined 10 SCI participants with a P300-based BCI system. In this study, participants had to spell the 5-character word “SPINE” by using a 36-character matrix (6 × 6 matrix layout) after 10 min of training (Jeon and Shin, 2015).

A recent study (Scherer et al., 2015) introduced a thought-based, row-column filtering correspondence board, emulating user-centered configuration standard for individuals with CP. More specifically, the left side of the screen contained the grid, while on the right side, feedback on the selected item was presented. Each row (item) was highlighted with a red-color box for certain time period. In the meantime the selection of a row (item) was visually reported back to the user by showing an animation sequence of the row (item) dissolving. Rows (items) were highlighted for 4 s with a 2-s break between the markers. It was shown that users became gradually more capable to communicate by using the proposed system.

Another study evaluated the possibility and usability of an assistive model operated by a P300-based BCI system in order to promote communication and environmental control of domestic appliances and applications to users with ALS (Schettini et al., 2015). Their results of the three experimental conditions [i.e., (i) P300-based BCI Speller, which contained a 6 × 6 matrix with 36 alphanumeric characters; (ii) the AT prototype operated by a conventional/alternative input device tailored to the specific end-user’s residual motor abilities; (iii) the AT prototype operated by a P300- based BCI] demonstrated that the effectiveness, adequacy and the end-user’s fulfillment did not vary within those three experimental states. However, the AT P300-based BCI system was less efficient and longer time was needed for correct selection. Moreover, in terms of the CA the participants’ performance was not satisfying.

In this vein, another study (Riccio et al., 2015) evaluated the impact of a hybrid control of a P300-based BCI technology (using both EEG and Electromyography) that was developed to operate an AT software. This study examined both able-bodied and people with motor impairment to assess effective communication and interaction with the environment. It was found that hybrid BCI might enable end-users to take advantage of some remaining muscular activity, which may not be fully reliable for properly controlling an AT device. Moreover, McCane et al. (2015) introduced also a 6 × 6 matrix where the participant had to “copy-spell” (35 letters) or “text–to–spell.” It is noteworthy that this study concluded that there is no significant correlation between level of severity and CA.

Finally, a combined approach tested a heterogeneous group of ALS participants in a study of a P300-BCI with an MI task and demonstrated for the first time that the quality of the control signals depend on the cognitive function of the participants and that behavioral dysfunction negatively affects P300 speller performance (Geronimo et al., 2016).

##### Web browsers

Another approach refers to BCI systems for Internet access. More recently a new solution of internet access, the “true web access,” was proposed by Mugler et al. (2010) where internet surfing could be successfully executed through a P300BCI browser. It was the first study to use real-life scenario for internet access by using the open-source technology of Mozilla’s Firefox. The browser was controlled by commands sent from the 6 × 6 P300 Speller system, while characters in the matrix could be used to select links in the browser window, to write emails or fill out forms. Their results showed that participants with ALS achieved a CA of 73% whilst healthy subjects 90% CA, and ITRs of 8.6 and 14.3, respectively. However, the positive response and acceptance of this useful tool was outlined by the ALS participants.

A recent work assessed the effectiveness, efficiency and user satisfaction in two spelling tasks, an email sending and an internet browsing task (Zickler et al., 2011) by testing a commercial “AT-software QualiWORLD” (QW) controlled by the P300 BCI with four end-users and three AT-experts. BCI stimulation was superimposed on the QW-interface thereby allowing the use of all functions of QW including an internet browser. The stimulation of the QW-interface and the web pages was performed by dots appearing directly next to the icon or link. Performance was high in all tasks and always above 70% accuracy. More importantly it was reported that the users were rather satisfied with the effectiveness of the device and did not mention any problems with the system’s performance.

##### Paint application

A different direction of a P300-BCI system is toward supporting users to paint (Münßinger et al., 2010). More specifically, the “Brain Painting” (color matrix and white-black matrix) included cells of a 6 × 8 matrix with symbols indicating color, objects, grid size, object size, transparency, zoom, and cursor movement. The experiment included three different tasks: spelling a preset sentence (copy-spelling), painting a preset picture (copy-painting), and painting an individual picture (free- painting). Despite the low ITR (ALS: 5.8, able-bodied: 8.57), the CA of both people with motor impairment and able-bodied was high (ALS: 79%, able-bodied: 92%). Moreover, the patients found the application extremely useful (Münßinger et al., 2010). However, this study showed that P300 amplitudes may be affected by illness severity as has been already mentioned in previous studies.

In addition, another P300-based BCI system used a “paint” application, achieving high performance levels (80% accuracy) in both free painting and copy painting conditions, whereas ITRs were rather low (4.47–6.65 bits/min) compared to other P300 applications. In this study it was stressed out that BCI-based painting is enhanced by using more symbols in the application interface, such as cursor movement, grid size, object shape, object size, color, transparency of color, zoom, undo/redo. The users reported that they were satisfied with the BCI Brain Painting application. In general, P300 Brain Painting application was effective and the end users with severe motor paralysis declared that they might utilize the suggested application in their everyday routine (Zickler et al., 2013). A recent study (Holz et al., 2015) reported the use of Brain Painting of 6 × 8 matrix including 48 tools for painting combined with a home-use P300-BCI application. After approximately 2 years of training a high accuracy of 70–90% was accomplished, while, most importantly, the end-users were highly satisfied with the BCI-Brain Painting system. This study highlighted the potential of using a BCI application independently by the users while promoting satisfaction and enjoyment to people with motor impairment.

##### Control of external devices

Another communication mode of P300-based systems is to control external devices. A recent study (Piccione et al., 2006) developed a BCI system that was tested by two groups of participants, where they had to select a specific path for a virtual object to reach the goal-point by using the P300 activity. They found differences between people with motor impairment and able-bodied regarding the P300 amplitudes, whereas without any training session both groups achieved quickly a successful trial and no improvement was found after training. As for the most impaired participants, their performance was worse than others, which indicates that P300 is affected when there is great impairment. On the other hand, it must be stressed out that the participants’ group was not homogenous since the authors examined five paralyzed participants with various impairments. It is worth noting that this is one of the few studies which have deployed such a system to people with motor impairment other than the common diseases (e.g., ALS and stroke). This study also highlights the fact that correct response without training by using P300 modulation is feasible as an endogenous response to a stimulus.

Furthermore, Mauro et al. (2011) compared two interfaces for controlling the movement of a virtual cursor on a monitor, and found that online CA was more than 70% both for able-bodied and ALS participants. Moreover, their results showed that ALS participants with residual motor abilities were able to maintain their attention, by controlling the movement of a virtual cursor on a monitor, similar to the able-bodied ones. Also, it was stressed that ALS participants could improve their performance and communication response with a P300-guided BCI. Another approach evaluated different visual P300 BCI systems with individuals with severe disabilities and their results revealed that P300-based BCI operation may be affected by the severity of the disease, whereas long periods of BCI execution revealed tiredness symptoms and a decrease in performance rates (Pires et al., 2012).

A recent work on this field tested a P300-based BCI system on participants with different motor and cognitive limitations, aiming at managing eight real domestic devices by means of 113 control commands (Corralejo et al., 2014). Ten out of 15 participants were able to properly execute the suggested apparatus for precision higher than 75%. Eight of them reached accuracy above 95%. Moreover, high ITRs, up to 20.1 bit/min were reached. As in McCane et al. (2015), it was suggested that the degree of motor impairment and the level of disease severity is not related to successful operation of a P300-based BCI tool (Corralejo et al., 2014).

In another study a BCI system decodes the user’s intentions and facilitates navigation, exploration and bidirectional communication with a robotic system that is remotely controlled (Escolano et al., 2010). More specifically, the system is consisted of a user station (patient environment) and a robot station (placed anywhere in the world), both remotely located and through the Internet to promote communication. Although the ITR was significantly low (7 bits/min) the CA was interestingly high (90–100%).

### Rehabilitation and Training Using EEG-Based BCI

One of the most significant and innovative applications for BCI technology concern rehabilitation systems that aim to support people with motor impairments to regain the lost motor control. To address the increasing challenge to deal with acquired disability, it is imperative that effective rehabilitation and treatment methodologies being developed for addressing each stage of after stroke recovery.

Many clinical BCI studies showed evidence for the feasibility and positive effect of MI-based BCI systems in combination with physiotherapy and robotic assistive orthotic devices for motor post–stroke recovery (Pfurtscheller et al., 2000; Grosse-Wentrup et al., 2011; Kasashima-Shindo et al., 2015; Nicolas-Alonso et al., 2015; Arvaneh et al., 2016). It is conjectured (Grosse-Wentrup et al., 2011; Ramos-Murguialday et al., 2013; Kasashima-Shindo et al., 2015; Nicolas-Alonso et al., 2015; Chaudhary et al., 2016) that this efficacy of BCI systems on motor rehabilitation is due to the underling mechanism of synaptic plasticity (Morris, 1999). In particular, the process of synaptic plasticity enables “the learning of new information and the acquisition of new skills, while the human brain is able to restore normal brain function, by affecting motor learning, for instance, by demanding close attention to a motor task or by requiring the activation or deactivation of specific brain signals” (Daly and Wolpaw, 2008). Various recent studies have shed light in the BCI-based rehabilitation training and motor retraining through real, virtual (Leeb et al., 2007) and augmented approaches and have managed to identify the characteristics of a MI- based BCI system for rehabilitation.

In this vein, it is suggested that there are two ways for paralyzed people to regain motor abilities by using BCI systems: (i) train patients to produce more reliable motor brain signals, and (ii) train patients to activate a device that assists movement by improving the motor function (Remsik et al., 2016). Even though people with acquired motor impairment often exhibit damaged cortex or disrupted motor connection integrity, (EEG)-based BCI methods are still capable of identifying meaningful improvement and gradual change. By relying on the capacity of residual motor neurons to trigger and facilitate appliance control, BCIs help to train persisting cortical connections to execute motor output of the motor-impaired limb (e.g., hand). In general, EEG-BCI has implications for the potential of recovery while it can be considered as an assistive solution to traditional physiotherapeutic approaches. The combined approach of BCI systems together with traditional physiotherapy appears to be very effective. A recent systematic review has demonstrated the benefits and the clinical efficacy of BCI systems in motor recovery of post-stroke patients during stroke rehabilitation process, in combination with traditional physiotherapy and robotic assistive orthotic devices (Remsik et al., 2016).

In another work, a tetraplegic participant gained control of a hand orthosis in order to improve his functionality of residual muscle activity and restore “hand grasp function” of the upper limb. This was achieved by regulating his SMR through motor imaging of his foot movement (Pfurtscheller et al., 2000). More specifically, MI of the hand movements (right or left) yielded generally moderate classification rates, whereas in the case of MI of foot movement, the classification performance increased gradually and significantly over time. Following the training procedure the participant was capable for a successful operation of the orthosis by closing the hand orthosis while imaging both-feet-movement and by opening the orthosis by imaging right-hand-movement, nearly error-free with CA close to 100%. This was achieved mainly by the ability to induce voluntarily specific beta oscillations close to the foot area. Since a stable performance of above 90% correct responses was reached, the patient started to practically use the orthosis to lift light-weighted objects.

Another study, evaluated the results of daily BCI training to beneficial effects of physiotherapy in patients with severe paresis (Ramos-Murguialday et al., 2013). During BCI-training patients were instructed to desynchronize SMR by imagining moving their severely impaired upper limb. Successful SMR control resulted in concurrent movements of the arm and hand orthoses in the experimental group, while in the control group participants received sham feedback, i.e., random movements of the robotic orthoses (not related with the ipsilesional SMR oscillations). This was the first study to present SMR-BCI intervention as a rehabilitative approach for a relatively large number (*N* = 32) of stroke survivors.

A recent study (Pichiorri et al., 2015) highlighted the importance of the currently used BCI technology in assisting MI practice, which notably contributes to improvement of motor functionality in subacute stroke patients with severe motor disabilities. More specifically, they used BCIs which can support rapid measure of brain activity generated by MI. The BCI-monitored MI practice as an additional intervention strategy to common rehabilitation care in 28 subacute stroke patients with severe motor deficits proved to be successful. The importance of this study lies in the fact that it was a randomized controlled trial and the first to demonstrate a clinical, pre–post improvement of the subacute stroke patients with detailed report about the underlying neurobiology.

Moreover, a recent study suggested to enhance motor recovery in people who survived a stroke through passive movement (PM) with a haptic robot and MI (Arvaneh et al., 2016). This approach included 3 runs of 40 trials each. More specifically, a cue was given for 2 s and the command to take action for 4 s, respectively, while the movement of the chosen hand was performed using the haptic knob robot (Passive Movement, PM) or Imaging movement of the paralyzed (left/right) hand (MI). The results revealed similar CA for both healthy participants and participants with motor impairment after stroke with hemiparesis. Moreover better CA of stroke participants (75%) than able-bodied (67.7%) was observed.

Finally, another study demonstrated the potential application of a MI task as a mechanism for stroke rehabilitation. They classified the participants into three sub-groups based on the different lesion locations in order to perform three different motor tasks (MI, passive motion, and active motion). The Motor Tasks included *active movements*, where grasping and supination movements with the affected hand were performed and *passive movements*, where a robotic device performed the movement. The MI was executed by imaging a movement, but the participants did not perform the physical movement. They found different β band EEG patterns in each patient group while two groups showed positive laterality coefficient (LC) values (LC of the ERD/ERS power of stroke patients is affected by brain damage) in the active and MI tasks. In these groups, the motor cortex was not directly damaged, therefore, a level of brain activation similar to that in able-bodied group was observed (Park et al., 2016). Thus, motor rehabilitation period differed depending on lesion location and these changes produced different patterns of neural activation in patients with chronic stroke on different lesion locations.

### Tabulated Overview of EEG-Related Studies

**Table 1** lists the reviewed works included in section “EEG-Based BCI Systems for People with Motor-Impairment” and summarizes the number of participants, the specific disease, the EEG-BCI application, and the EEG-BCI modalities used in each one of them. The studies are tabulated in chronological order for each one of the EEG-BCI modalities.

**Table 1:** Summary of EEG-BCI studies for communication, control and rehabilitation involving people with motor impairment.

Study	Participants	Disease	EEG-BCI application	EEG-based neurophysiological input
Birbaumer et al., 1999	Two participants	ALS	TTD system	SCP
Birbaumer et al., 2000	Five participants	ALS	TTD system	SCP
Kubler et al., 2001	Two participants	ALS	TTD system	SCP
Hinterberger et al., 2003	One participant	ALS	TTD system	SCP
Neumann and Birbaumer, 2003	Five participants	ALS	TTD system	SCP
Neumann et al., 2003	One participant	ALS	Visual feedback	SCP
Karim et al., 2006	One participant	ALS	Web browsers	SCP
Pfurtscheller et al., 2000	One participant	SCI	Rehabilitation	Motor imagery and SMR
Neuper et al., 2003	One participant	CP	Graz-BCI	Motor imagery and SMR
Krausz et al., 2003	Four participants	Paraplegia	Graz-BCI	Motor imagery and SMR
Wolpaw and McFarland, 2004	Four participants (two people with motor impairment and two able-bodied)	SCI	Cursor movement systems	Motor imagery and SMR
Kübler et al., 2005	Four participants	ALS	Cursor movement systems	Motor imagery and SMR
Kauhanen et al., 2007	Six participants	Five SCI, one G-B	Game application	Motor imagery and SMR
Leeb et al., 2007	One participant	SCI	Virtual environments	Motor imagery and SMR
Bai et al., 2008	11 participants (nine able-bodied and two people with motor impairment)	One Stroke, one ALS	Game application	Motor imagery and SMR
Cincotti et al., 2008a	Six participants (five able-bodied and one people with motor impairment)	SCI	Cursor movement systems	Motor imagery and SMR
Cincotti et al., 2008b	28 participants (14 able-bodied 14 people with motor impairment)	Eight with SMA II/III, six with DMD	Control of external devices	Motor imagery and SMR
McFarland et al., 2008	Six participants (four able-bodied two people with motor impairment)	SCI	Cursor movement systems	Motor imagery and SMR
Babiloni et al., 2009	20 participants (14 able-bodied and six people with motor impairment)	DMD	Control of external devices	Motor imagery and SMR
Conradi et al., 2009	Seven people with motor impairment participants	SCI	Control of external device	Motor imagery and SMR
Holz et al., 2013	Four participants	One tetra paresis, one CP, two cerebral bleeding	Game applications	Motor imagery and SMR
Ramos-Murguialday et al., 2013	32 participants	Stroke	Rehabilitation	Motor imagery and SMR
Leeb et al., 2015	29 participants (9 people with motor impairment participants and 10 able-bodied)	Six SCI, two myopathy, one DMD	Control of external devices	Motor imagery and SMR
Pichiorri et al., 2015	28 participants (14 BCI MI training group and 14 no-BCI MI training group)	Stroke	Rehabilitation	Motor imagery and SMR
Geronimo et al., 2016	40 participants (25 people with motor impairment and 15 able-bodied)	ALS	Control of external device	Motor imagery, SMR, and P300
Arvaneh et al., 2016	22 participants (six people with motor impairment and 16 able-bodied)	Stroke	Rehabilitation	Motor imagery and SMR
Park et al., 2016	32 participants (20 people with motor impairment and 12 able-bodied)	Stroke	Rehabilitation	Motor imagery and SMR
Sellers and Donchin, 2006	Six participants (three able-bodied and three people with motor impairment)	ALS	Speller systems	P300
Piccione et al., 2006	12 participants (five people with motor impairment and seven able-bodied)	ALS, MS, GB, Stroke, SCI	Control of external devices	P300
Hoffmann et al., 2008	Nine participants (five people with motor impairment and four able-bodied)	CP, MS, ALS, TBI-SCI, Post-anoxic encephalopathy	Speller systems	P300
Nijboer et al., 2008	Six participants	ALS	Speller systems	P300
Kübler et al., 2009	Four participants	ALS	Speller systems	P300
Kaufmann et al., 2013	25 participants (16 able-bodied and 9 motor-impaired)	Two SMA II, four ALS, two SBMA, one MD	Speller systems	P300
Escolano et al., 2010	One participant	ALS	Web browsers	P300
Mugler et al., 2010	13 participants (10 able-bodied and 3 people with motor impairment participants)	ALS	Web browsers	P300
Münßinger et al., 2010	26 participants (20 able-bodied and three people with motor impairment)	ALS	Paint application	P300
Mauro et al., 2011	Eight participants (four able-bodied and four people with motor impairment)	ALS	Control of external devices	P300
Nam et al., 2012	18 participants (nine able-bodied and nine people with motor impairment)	Three ALS, six CP	Speller systems	P300
Pires et al., 2012	14 participants	Five CP, one DMD, one SCI, seven ALS	Control of external devices	P300
Zickler et al., 2011	Seven participants (four people with motor-impairment and 3 AT experts)	One ALS, one SMA III, three DMD	Web browsers	P300
Zickler et al., 2013	Four participants	Two ALS, one stroke, one DMD	Paint application	P300
Corralejo et al., 2014	15 participants	Six Spastic CP, one A-CM, two SCI, three TBI, one MS, one Neurofibromatosis and severe kyphoscoliosis, one Extrapyramidal syndrome, dystonia and parkinsonism	Control of external devices	P300
Scherer et al., 2015	14 participants	Dystonic/Spastic CP	Speller systems	P300
Holz et al., 2015	Two participants	ALS	Paint application	P300
Jeon and Shin, 2015	Ten participants	SCI	Speller systems	P300
Schettini et al., 2015	Eight people with motor impairment participants	ALS	Speller systems	P300
Riccio et al., 2015	11 participants (eight able-bodied and three people with motor impairment)	Two Stroke, one ALS	Speller systems	P300
McCane et al., 2015	28 participants (14 people with motor impairment and 14 able-bodied)	ALS	Speller systems	P300


## Discussion

The aim of this review is to identify and synthesize findings on the grounds of non-invasive EEG-based BCI systems. We presented published studies which promote communication and control of appliances for people with motor impairment and approaches which applied adaptable rehabilitation strategies concerning the modern restorative physiotherapy. We overviewed evidence regarding the effective use, modalities and useful applications of non-invasive EEG-BCI systems from studies including people with motor impairment. In particular, BCI systems of studies published the last two decades were reviewed, discussing the added value of this novel technology and highlighting the important role of BCIs in life of people with motor impairment. These studies involved applications of BCI systems in fields such as medical and clinical applications, control of wheelchair, games and entertainment (e.g., painting), communication, rehabilitation and environmental control.

Different control mechanisms have been used to assess the CA and ITR of the system and the ability of the user to modulate brain patterns. However, contextual factors that can influence the performance in real-life scenarios have not been discussed thoroughly. The majority of the studies have recruited participants with adult-onset ALS, while most of them were at severe level of paralysis such as LIS. In studies focusing on rehabilitation, the majority of participants were tetraplegic with SCI at cervical vertebrae. The most significant goals that have driven BCI research over the last decades have been stressed within the studies. From the reviewed studies it is evident that each one of the three EEG-based modalities (i.e., SCP, SMR, and P300), used in non-invasive BCIs, comprise a promising solution for EEG-BCI system realization.

Moreover, many studies have successfully addressed the problem of user’s training duration, which has been essentially decreased and has led to additional broad BCI systems deployed in the everyday routine of people with motor impairment, such as word processing, use of browsers, sending and reading of emails (Zickler et al., 2011), control of devices, such as wheelchair and domestic appliances. In Holz et al. (2013) a SMR based BCI for entertainment purposes, the so-called “Connect-Four,” which is a strategic game with two competitive players demonstrated that despite the low effectiveness of participants compared with similar P300 BCI systems, such entertainment applications are greatly accepted by the end-users.

Moreover, SMR-based games (Kauhanen et al., 2007; Bai et al., 2008) indicated that people with motor impairment show reliable performance and a successful BCI operation. In Kauhanen et al. (2007) three out of six subjects learned to control a BCI after training, where a great advantage was that participants received feedback and could change their strategy in response to the feedback. In Bai et al. (2008) both patients were able to use the proposed SMR-BCI game system with high performance as well. Both studies are highlighting the adaptability of the systems and the general acceptance of game applications by people with motor impairment. However, in SMR BCI applications a main disadvantage is that although ERD/ERS is observed in the majority of participants, some subjects (even able-bodied) may have no detectable ERD/ERS components (on the contrary P300 component is always observed). Initially, these BCI approaches for entertainment were typically not at high priority in the field of BCI research due to the fact that BCI research has mainly focused on applications to address communication and independency through assistive technological ways (i.e., spelling devices, control of external devices). Nevertheless, game oriented solutions seem to be really promising since they use additional assistive tools while enhancing participant’s motivation.

Despite high performance rates in some reviewed studies there are various others that showed extremely low performance in terms of both CA and ITR. Moreover, reported BCI studies involving both able-bodied individuals and those with severe disabilities have pointed delays in reaction time, low ITR and worse CA of people with motor-impairment (Bai et al., 2008; Escolano et al., 2010; Mugler et al., 2010; Nam et al., 2012; Pires et al., 2012; Holz et al., 2013; Jeon and Shin, 2015). Moreover, low ITRs of current BCI systems do not allow for general conclusions regarding the effective use of BCI on a daily basis. Also, except for few cases (Vansteensel et al., 2016), the majority of BCI systems and applications are mainly used in a research environment (research laboratory etc.) and cannot be successfully utilized in patients homes for continuous and everyday use, as they need adaptation and fixation during the operation.

P300 exhibits higher ITRs and does not need training but is greatly affected by the level of severity of the disease. Nevertheless, many studies have also shown that even patients in the LIS can use a P300 BCI for long term periods (Sellers et al., 2010; Holz et al., 2015). However, in terms of ITR, able-bodied group reached higher maximum bit rates than disabled subjects in almost all studies of P300-BCI (Escolano et al., 2010; Mugler et al., 2010; Mauro et al., 2011; Vaughan et al., 2011; Nam et al., 2012; Pires et al., 2012). Moreover, in some studies the patients did not even complete the experimental process (Mauro et al., 2011). In addition, in most cases the most severely paralyzed participants seem not to be able to operate successfully the EEG based BCI system (Pires et al., 2012; Pasqualotto et al., 2015). These results indicate that (i) P300 may be affected by level of severity, (ii) participants have worse performance during the sessions due to a “habitual effect” (Ravden and Polich, 1998, 1999; Kececi et al., 2006).

The reviewed articles largely focused on individuals with adult-onset disabilities. It is unclear if the findings of these studies could be generalized to individuals with congenital disabilities, who often have never experienced any terms of communication or motion. For example, to the best of our knowledge, BCI systems which use SMR for system operation rely on MI of upper and lower limbs and have not been tested with individuals who never experienced voluntary control of their movements (Moghimi et al., 2013).

Albeit slow, in the majority of the studies SCP speller (namely the TTD) was around 1 letter per minute and satisfied the requirements for a successful BCI system, (Birbaumer et al., 2000; Hinterberger et al., 2003). Although the course of the SCP shifts of participants who used the TTD remained stable over time, a huge disadvantage of BCIs that demand “self-control” of an EEG component is that the user must undergo long-term preparation and extensive training for several weeks so as to gain the level of CA needed to utilize, e.g., “brain-controlled cursor movement” for communication (Birbaumer et al., 2000; Hinterberger et al., 2003).

One limitation of our review is that we do not provide a comparison of the performances of different P300, SMR, and SCP classifiers for healthy and people with motor impairment and thus do not investigate to what extent this fact plays a key role in BCI research as other studies and systematic reviews have already reported (Manyakov et al., 2011; Abdulkader et al., 2015). Thus, since a classifier comparison has never been performed on participants, it remains an open question what might be the best classifier in each case. This is definitely a critical inquiry since the brain activation and responses from people with motor impairment and able-bodied participants might be different (Nam et al., 2012).

Future and on-going studies should try to address the fact that EEG patterns change based on the user’s learning ability and multiple adjustments of the classifiers can result in better communication performance. This suggests the development of parameter estimation processes and the development of adaptive classification frameworks. Thus, a technical expert who will adjust the BCI system’s parameters is essential to be on-site. The practical problem is that it is not possible for the ‘BCI-expert,’ to visit repeatedly the user for training sessions. Also as EEG signals reflect the dynamics of the interaction between the user and the BCI systems, error-related potentials (Chavarriaga et al., 2014) may be a useful solution as an auto-correction strategy.

Moreover, a development of a BCI technology that does not replace but complement existing therapies is a novel and promising field. Studies in stroke patients have shown that, with a motor relearning intervention, EEG features change in parallel with improvement in motor function and that sensorimotor rehabilitation using BCI training and MI may improve motor function after central nervous system injury. Taking into account all the above mentioned pieces of evidence, there is a strong assumption that BCIs can eventually promote the independence through novel communication techniques and promote motor rehabilitation of patients with NMD.

## Conclusion and Future Perspectives

The most recent progress alongside BCI research suggest that novel and innovative multimodal developments or combined solutions using EEG- BCI and other assistive technologies together (e.g., eye-trackers) might be made imminent in the close future (EU Horizon 2020 project MAMEM, No. 644780: Multimedia Authoring and Management using your Eyes and Mind under contract, Coordinator: CERTH, 2015–2018^2^)^3^. These accomplishments and the possibility of new BCI systems have clearly provided with a noteworthy “boost” of BCI research including a large number of researchers from different disciplines, e.g., neuroscientists, physicians, electrical engineers, and clinical rehabilitation professionals, among others. Research interest in the field of BCI systems is expected to increase and BCI design and development and will most probably continue to bring benefits to the daily lives of people with motor impairment. In the near future, BCI systems may therefore become a new mode of human–machine interaction with levels of everyday use that are similar to other current interfaces (Corke, 2007; Gajwani and Chhabria, 2010). Moreover to address the issue of extensive training for self-regulation of SMR, more enjoyable solutions such as Virtual Reality or Painting (Holz et al., 2015) could be used. These approaches re-enable patients to be creatively active and consequently promote feelings of happiness, self-esteem and well-being, and promote better QoL. Also, as the goal of future studies should be the demonstration of a long-term beneficial impact of BCI technology on functional recovery and motor rehabilitation, extensive randomized controlled trials are required.

The development of novel BCIs raises new hopes for the motor rehabilitation of people with motor impairment (Daly and Wolpaw, 2008). Therefore, combination of MI to improve efficacy of physiotherapy in stroke rehabilitation will be a promising solution. However, the majority of current published works are basically proof of concept studies with no clinical based evidence of daily use by people with motor impairment. Thus, the acceptability and usability of future developed EEG-BCI systems might be two important issues depending on the size, complexity of the EEG device and successful operation that should be taken into consideration in the future. Nevertheless, BCI systems have already demonstrated their efficacy and reliability. In sum, as was reported in Wolpaw et al. (2002): “non-muscular communication and control is no longer merely speculation while at the same time, the reality does not yet match the fantasy.”

## Author Contributions

IL wrote, formatted, and submitted the paper. SN wrote, formatted, and corrected it. PP formatted the paper and made comments and corrections. IK made comments and corrections and approved the paper. MT made the final correction and approved the paper.

## Conflict of Interest Statement

The authors declare that the research was conducted in the absence of any commercial or financial relationships that could be construed as a potential conflict of interest.

## Footnotes

1 https://www.naturalpoint.com/smartnav/

2 http://www.mamem.eu/

3 MAMEM’s goal is to integrate motor-impaired people back into society by increasing their potential for communication and exchange in leisure (e.g., social networks) and non-leisure context (e.g., workplace) by using EEG-based BCI approach.

## Abbreviations

A-CM: Arnold-Chiari malformation
ALS: amyotrophic lateral sclerosis
BCI: brain–computer interface
CA: classification accuracy
CBP: checkerboard paradigm
CCD: cortical current density
CLIS: complete locked-in state
CP: cerebral palsy
CRR: correct response rate
DMD: Duchenne muscular dystrophy
EEG: electroencephalogram
ERD: event-related de-synchronization
ERP: event-related potential
ERS: event-related synchronization
ITR: information transfer rate
LC: corresponding laterality coefficient
LIS: locked-in state
MI: motor imagery
MS: multiple sclerosis
NMD: neuromuscular disorders
RCP: row–column paradigm
SCI: spinal cord injury
SCPs: slow cortical potentials
SMA II/III: spinal muscular atrophy type II/III
SMR: sensorimotor rhythms
Spastic CP: spastic cerebral palsy
TBI: traumatic brain injury
TTD: thought translation device
VE: virtual environment
